# Morita–Baylis–Hillman modifications on hydroformylated (*R*)-limonene

**DOI:** 10.1098/rsos.251277

**Published:** 2025-11-26

**Authors:** José Ribeiro Gregório, Ricardo Gomes da Rosa, Bruno Mascarenhas Oliveira, Celso Vataru Nakamura

**Affiliations:** ^1^ Departamento de Química Inorgânica, Universidade Federal do Rio Grande do Sul, Porto Alegre, Rio Grande do Sul, Brazil; ^2^ Departamento de Ciências Básicas da Saúde, Universidade Estadual de Maringa, Maringa, Paraná, Brazil

**Keywords:** (*R*)-limonene, hydroformylation, Morita–Baylis–Hillman reaction, antitumoural activity

## Abstract

We report the sequential hydroformylation of (*R*)-limonene followed by Morita–Baylis–Hillman (MBH) coupling to give two new MBH adducts derived from methyl acrylate (adduct 1, 32% isolated yield) and acrylonitrile (adduct 2, 95% isolated yield). The compounds were characterized by ^1^H- and ^13^C-NMR, IR spectroscopy and elemental analysis. Biological screening against protozoa, bacteria, fungi and human cell lines revealed modest antiprotozoal and antimicrobial activity, and the ester adduct (adduct 1) showed the most promising antiproliferative activity (CC_50_ values: HT-29 = 211.4 ± 49.1 μg ml^−1^; PC-3 = 48.0 ± 2.6 μg ml^−1^; HeLa = 37.9 ± 6.7 μg ml^−1^; HACAT = 53.3 ± 3.1 μg ml^−1^). These results introduce a new set of terpene-derived MBH adducts with preliminary antitumoural potential and encourage further optimization and mechanistic studies.

## 1. Introduction

Vegetable oils constitute an important class of renewable feedstocks for the chemical industry, but they comprise chemically distinct categories. On the one hand, triglyceride-based oils are composed mainly of fatty acid esters of glycerol obtained from seeds and fruits, and serve primarily as sources of biodiesel, lubricants and surfactants. On the other hand, essential oils are volatile mixtures of mono- and sesquiterpenes produced by steam distillation or cold pressing of plant material. These terpene-rich essential oils are valuable platforms for functionalization due to their structural diversity and availability from biomass. The present work focuses on transformations of (*R*)-limonene, the principal constituent of citrus essential oils.

(*R*)-Limonene is a renewable, chiral terpene with two non-conjugated double bonds that can undergo selective transformations such as hydroformylation. This reaction, discovered in 1938 by Otto Roelen, involves the addition of a formyl group and a hydrogen atom to a carbon–carbon double bond in the presence of transition-metal catalysts, yielding aldehydes with an extra carbon atom relative to the starting olefin [1]. It is a clean method with 100% atom economy [2] (all reagent atoms are incorporated into the final product) and can be performed in the presence of several functional groups, such as nitriles, amides and esters [3–10]. This process is among the most versatile carbon–carbon bond-forming reactions in industry and has recently been extended to renewable substrates such as terpenes and unsaturated fatty esters. Several studies have demonstrated the hydroformylation of (*R*)-limonene under mild rhodium-catalysed conditions, yielding functionalized aldehydes that can serve as intermediates for fine chemicals and pharmaceuticals.

**Scheme 1.**
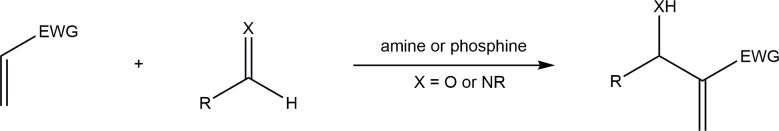
Morita–Baylis–Hillman reaction.

The Morita–Baylis–Hillman (MBH) reaction, in turn, is a carbon–carbon bond-forming process between an activated alkene and an aldehyde under nucleophilic catalysis (see Scheme 1).

The reaction produces allylic alcohols bearing densely functionalized β-substituted carbon frameworks with potential added value and bioactivity [11–20]. When combined with hydroformylation, MBH chemistry offers a powerful route to construct highly functionalized derivatives directly from renewable terpenes. The combination of these two atom-economical reactions aligns well with current goals of green and sustainable chemistry. Beyond their synthetic interest, Morita–Baylis–Hillman adducts derived from terpenes represent a versatile class of multifunctional intermediates. Their densely functionalized allylic frameworks make them valuable precursors for further derivatization, such as the conversion of nitrile or ester substituents into amides, acids or alcohols. In medicinal chemistry, MBH adducts have attracted attention as scaffolds for anticancer, antiparasitic and antimicrobial leads, due to their ability to engage in hydrogen bonding and Michael-type interactions with biological targets. In addition, these adducts can serve as building blocks for fine chemicals, agrochemicals and polymeric materials, linking renewable terpene feedstocks to high-value applications. Recent reviews summarize the expanding scope and bioactivity of MBH derivatives [21,22].

Since our group has long been working on transformations of (*R*)-limonene [18,23–27], the present study reports the sequential hydroformylation of (*R*)-limonene followed by MBH coupling with methyl acrylate and acrylonitrile, yielding two novel adducts. The products were characterized by NMR and IR spectroscopy and elemental analysis, and evaluated for biological activity against protozoa, fungi, bacteria and tumour cell lines. This work demonstrates the feasibility of generating bioactive terpene-derived MBH adducts and highlights the potential of combining hydroformylation and MBH methodologies in the sustainable transformation of essential oil constituents.

## 2. Material and methods

### 2.1. Material

RhH(CO)(PPh_3_)_3_ was prepared according to the literature [28] with a 75% yield. IR and ^1^H-NMR spectra were as expected. (*R*)-Limonene (Aldrich, 97%) was used as received. Other reagents were purchased from Aldrich (methyl acrylate, 99%; triphenylphosphine, 99%; and DABCO (1,4-diazabicyclo[2.2.2]octane), 99%), Acros (acrylonitrile, 99%) and Merck (triethylamine, 99%). The gases H_2_ and CO employed in hydroformylation were both of 99% purity and purchased from White Martins.

### 2.2. Hydroformylation procedure

The reactions were performed in a 100 ml steel reactor equipped with a thermocouple, under magnetic stirring, and without solvent. In a typical reaction, the substrate (51.4 mmol), the catalytic precursor (23.0 mg, 0.0250 mmol) and triphenylphosphine (45.7 mg, 0.174 mmol) were transferred to the reactor, which was sealed and purged three times with a mixture of carbon monoxide and hydrogen (2 : 1). The reactor was then pressurized to 40 bar, heated to 100°C and the reaction time counted from the moment at which this temperature was reached. After 6 h, the reactions were stopped by cooling to room temperature with a water bath and depressurization. The products (a mixture of starting oil and its hydrogenated and hydroformylated analogues) were separated, dried and analysed by NMR. It was found that a silica gel column could not be used for separation, as a reaction occurs between the aldehyde formed and the silane groups, retaining the product in the column. For this purpose, a ball oven was used, and GC was used to determine the individual concentration of each component (remaining (*R*)-limonene and its derivatives, hydrogenated and hydroformylated). The GC calibration was performed according to Tranchant *et al.* [29].

### 2.3. Morita–Baylis–Hillman reaction

To a Schlenk flask containing hydroformylated (*R*)-limonene and DABCO (1 : 1 molar ratio) under argon, methyl acrylate (or acrylonitrile) was added in a 3.5 : 1 molar ratio at room temperature. After 3 days, the mixture was eluted on silica gel (hexane : ethyl acetate, 4 : 1, volume ratio), the solvent was evaporated in a rotatory evaporator and then dried under vacuum, yielding the adducts. All products were characterized by IR spectroscopy, CHN analysis, and ^1^H- and ^13^C-NMR, confirming the expected structures.

### 2.4. Characterization of products

#### 2.4.1. Hydroformylated (*R*)-limonene

**Figure 1.**
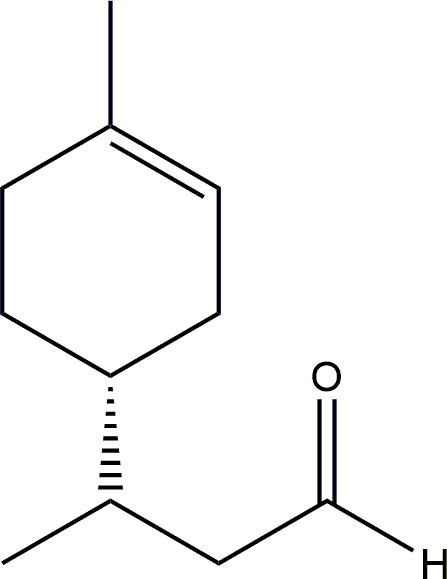
Hydroformylated (*R*)-limonene.

Colourless oil, 79% isolated yield. Formula C_11_H_18_O, molecular mass 165.55 g. ^1^H-NMR (400 MHz, CDCl_3_) δ = 9.77 (m, 1H), 5.38 (m, 1H), 2.52 (m, 1H), 2.24 (m, 1H), 2.03 (m, 4H), 1.74 (m, 2H), 1.64 (s, 3H), 1.46 (m, 1H), 1.26 (m, 1H), 0.95 (t, 3H). ^13^C-NMR (100 MHz, CDCl_3_) δ = 203.1, 133.9, 120.4, 48.6, 38.5, 32.3, 30.7, 29.1, 26.7, 23.4, 17.1 (see figure 1).

#### 2.4.2. (*R*)-Limonene adduct with methyl acrylate (adduct 1)

**Figure 2.**
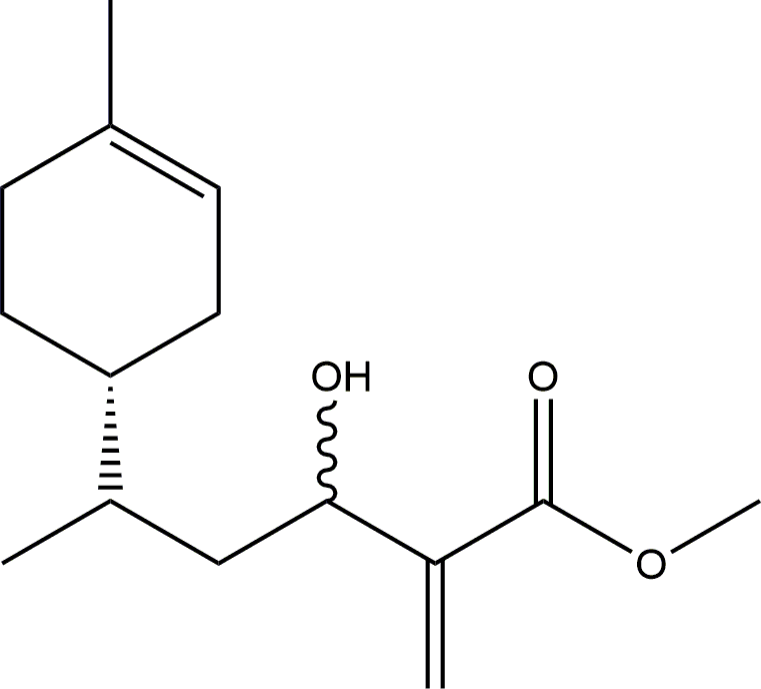
(*R*)-Limonene adduct with methyl acrylate.

Colourless oil, 32% isolated yield. Formula C_15_H_24_O_3_, molecular mass 252.35 g. ^1^H-NMR (400 MHz, CDCl_3_) δ = 6.22 (d, 1H), 5.82 (m, 1H), 5.37 (m, 1H), 4.47 (m, 1H), 3.78 (s, 3H), 1.95 (m, 3H), 1.75 (m, 3H), 1.63 (s, 3H), 1.43 (m, 5H), 0.89 (m, 3H). ^13^C-NMR (100 MHz, CDCl_3_) δ = 167.0, 143.5, 133.9, 124.4, 120.9, 69.3, 51.9, 40.9, 39.1, 33.4, 30.8, 29.1, 25.5, 23.4, 16.0. Principal IR peaks: (cm^−1^): 3453, 2958, 2914, 1718, 1629, 1436, 1290, 1157. Elemental analysis: Calculated C (71.39%), H (9.59%). Experimental C (70.83%), H (9.44%) (see figure 2).

#### 2.4.3. (*R*)-Limonene adduct with acrylonitrile (adduct 2)

**Figure 3.**
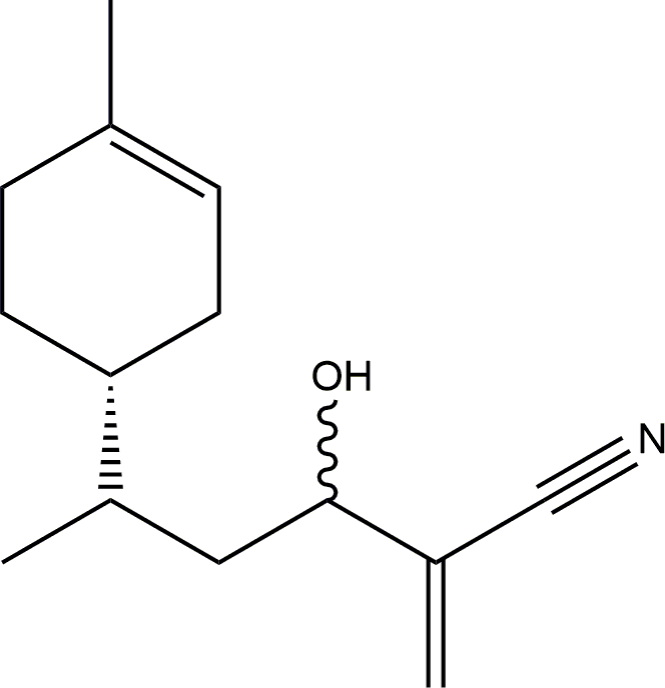
(*R*)-Limonene adduct with acrylonitrile.

Yellow oil, 95% isolated yield. Formula C_14_H_21_NO, molecular mass 219.33 g. ^1^H-NMR (400 MHz, CDCl_3_) δ = 6.00 (m, 2H), 5.37 (m, 1H), 4.31 (m, 1H), 2.15 (m, 1H), 1.96 (m, 3H), 1.82 (m, 2H), 1.68 (m, 2H), 1.64 (s, 3H), 1.51 (m, 2H), 1.28 (m, 1H), 0.92 (m, 3H). ^13^C-NMR (100 MHz, CDCl_3_) δ = 134.0, 129.5, 127.7, 120.7, 117.3, 70.2, 40.2, 38.9, 33.2, 30.7, 29.1, 23.4, 15.7. Principal IR peaks: (cm^−1^): 2965, 1653, 1413, 1261, 1094, 1010, 910, 867, 802, 739. Elemental analysis: Calculated C (76.67%), H (9.65%), N (6.39). Experimental C (74.30%), H (9.15%), N (8.17%) (see figure 3).

### 2.5. Biological evaluation

Adducts 1 and 2 were tested against three pathogen groups: protozoa, tumoural cells and other microorganisms, such as fungi and bacteria. A different methodology was adopted for each group to determine which group the adducts possess the more extensive biological activity and, therefore, are most promising against them.

#### 2.5.1. Evaluation of antiprotozoal activity

Promastigotes from *Leishmania amazonensis* in their log growing phase (48 h) and epimastigotes from *Trypanosoma cruzi* (96 h) in concentrations of 1 × 10^6^ cells ml^−1^ were dispensed in 96-well sterile plates in the presence of increasing concentrations of the adducts (1 to 500 μg ml^−1^). After 72 h incubation at 25°C or 96 h at 28°C for promastigotes and epimastigotes, respectively, the activity was determined through reduction methodology of XTT (2,3-bis(2-methoxy-4-nitro-5-sulfophenyl)-2*H*-tetrazolium-5-carboxanilide). For that, 50 μl well^−1^ of a 0.5 mg ml^−1^ XTT and 0.06 mg ml^−1^ PMS (1-methoxy-5-methylphenazinium methyl sulfate) solution were added and incubated in the absence of light at 25 or 28°C according to the parasite. After 4 h, the absorbance at 450 nm was read using a spectrophotometer. The average of three independent results was plotted as concentration versus percentage of growth inhibition to obtain the concentration capable of inhibiting 50% of the parasite growth compared with the control sample (IC_50_) [30].

#### 2.5.2. Evaluation of antitumoural activity

For the assessment of cytotoxic activity, suspensions of HT-29 (colon carcinoma), HeLa (cervical carcinoma), PC-3 (prostate carcinoma) and HACAT (immortalized human keratinocytes) cells at concentrations of 2.5 × 10^5^ cells ml^−1^ were disposed in sterile 96-wells plates and incubated for 24 h at 37°C and 5% CO_2_ tension. The supernatant was removed after this period, which was necessary for the adhesion and multiplication in the wells. Increasing concentrations (1 to 500 μg ml^−1^) of the products to be tested were added. Results were obtained after 48 h incubation under the same previously cited conditions (reduction of MTT, 3-(4,5-dimethyltiazol-2-yl)-2,5-diphenyltetrazolium bromide). After the culture media were removed, the wells were washed with PBS (phosphate-buffered saline solution). Subsequently, 50 μl of MTT (2 mg ml^−1^ in PBS) was added and the mixture was incubated in the dark at 37°C. After 4 h, 150 μl of DMSO was added to solubilize the crystals formed from MTT metabolized by the mitochondrial enzymes of viable cells, followed by reading at 570 nm using a spectrophotometer. The average of the results of three independent experiments was plotted on a graph relating the concentration of the compound and the percentage of cell contamination to calculate the concentration for which 50% of the cells are contaminated with the carcinoma in question, always concerning a control sample (CC_50_) [31].

#### 2.5.3. Evaluation of antimicrobial and antifungal activity

**Scheme 2.**
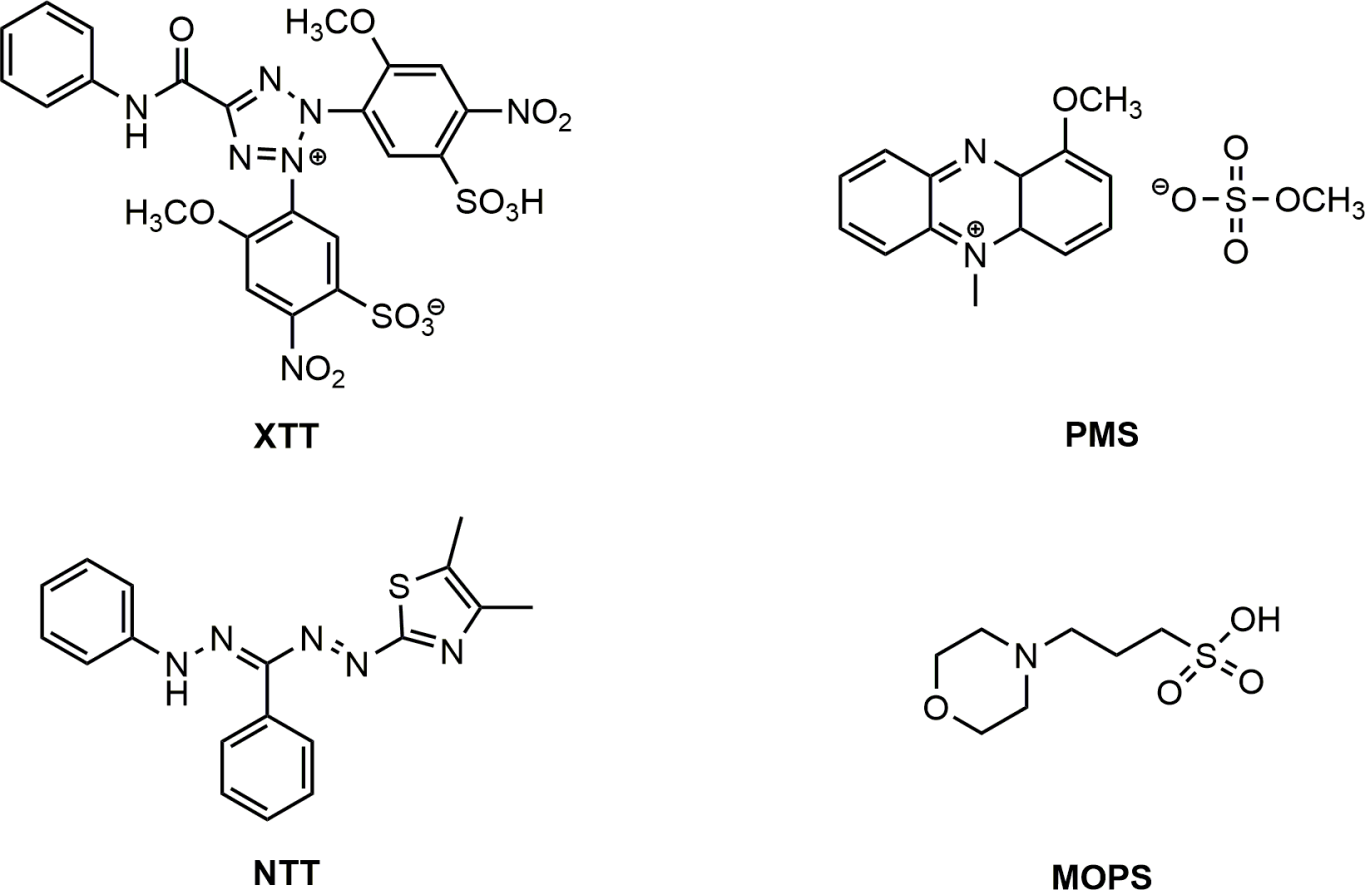
Structures of XTT, PMS, MTT and MOPS.

The microorganisms used in the tests were the bacteria *Escherichia coli* and *Staphylococcus aureus*, grown in Mueller–Hinton broth (MHB) at 37°C. The yeasts *Candida albicans* and *Candida tropicalis* were also used in the tests, both cultivated in Saboraud Dextrose (SDB) at 37°C. Before each experiment, the microorganisms were grown in the respective broths at 37°C for 24 h. For the evaluation of microbial activity, 96-well plates were used, in which 100 μl of MHB was distributed in each well, followed by the addition of 100 μl of the compounds diluted in HMB at a concentration of 2000 μg ml^−1^ (to have 1000 μg ml^−1^ in the first well), proceeding with serial dilution (1 : 2), homogenizing and transferring 100 μl to the next well until the last one. The microbial suspensions were standardized in 0.9% (w/v) NaCl solution, diluted 1 : 10 in MHB, and finally, 5 μl of the inoculum was added to each well of the plate. Positive controls (wells without compound) and negative controls (wells without extract and inoculum) were also performed. The plates were incubated at 37°C for 24 h. For yeasts, the MIC (minimum inhibitory concentration) was determined in the same way, differing using Rosewell Park Memorial Institute (RPMI) 1640 culture medium, pH 7.0, buffered with 0.165 mol l^−1^ 3-(*N*-morpholine)propanesulfonic acid (MOPS) buffer and the microbial suspensions were standardized in dilution 1 : 100. Then 5 μl was added to each well of the plates and incubated at 37°C for 48 h. After incubation, the bactericidal minimal concentration (BMC) and fungicidal minimal concentration (FMC) were determined using the subculture technique in Agar Muller Hinton and Agar Sabouraud for bacteria and yeasts, respectively. For this, 10 μl was removed from each well showing growth inhibition and from the positive control (well with visible growth), and the mixture was transferred to the agar plate and incubated at 37°C for 24 h. MIC is defined as the lowest concentration of an agent sufficient to inhibit the visible growth of the microorganism used in the sensitivity test. BMC or FMC is the lowest concentration that results in microbial death. For the analysis of the results, the antimicrobial activity was considered suitable for MIC less than 100 μg ml^−1^, moderate for MIC between 100 and 500 μg ml^−1^, weak for MIC between 500 and 1000 μg ml^−1^, and inactive for MIC greater than 1000 μg ml^−1^ [32] (see Scheme 2).

## 3. Results and discussion

### 3.1. Hydroformylation of (*R*)**-**limonene

**Scheme 3.**
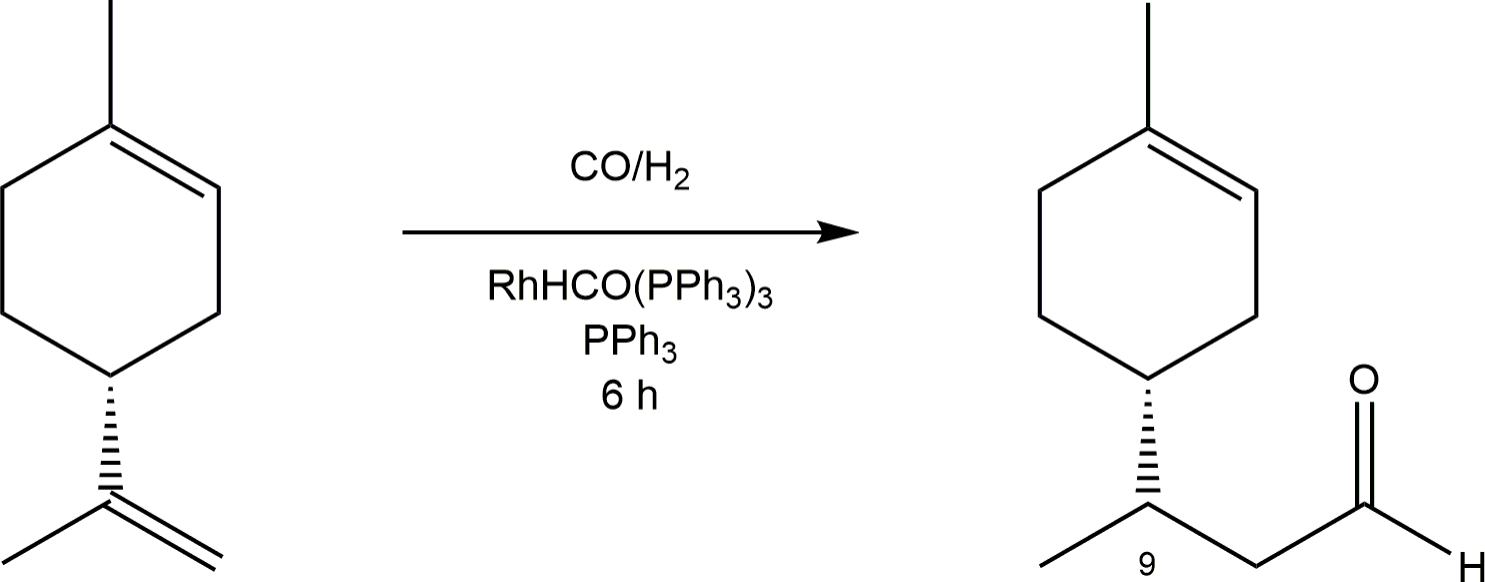
Hydroformylation of (*R*)-limonene.

This terpene has been widely employed as a starting olefin in several studies conducted by our research group [18,23–27]. Following the isolation of each product, hydroformylated (*R*)-limonene was characterized using both ¹H NMR and ¹³C NMR spectroscopy to confirm its formation. The reaction (Scheme 3), along with the spectral assignments (see §2), highlights the regioselectivity of the process.

The ¹³C NMR spectrum showed duplicated signals throughout, which can be attributed to the formation of two diastereomers arising from the stereogenic centre at carbon 9.

### 3.2. Morita–Baylis–Hillman reactions of the hydroformylated (*R*)**-**limonene

Using the hydroformylated product, several MBH reactions were carried out with the acrylic reagents methyl acrylate and acrylonitrile to optimize various reaction parameters, including reaction time, the acrylic reagent/DABCO/hydroformylated (*R*)-limonene ratio, reaction temperature and the need for an inert atmosphere. To the best of our knowledge, none of the derivatives presented herein has been previously reported.

The first observation at this stage was the confirmation of the need for an inert atmosphere, as aldehyde decomposition in air competes with adduct formation.

**Scheme 4.**
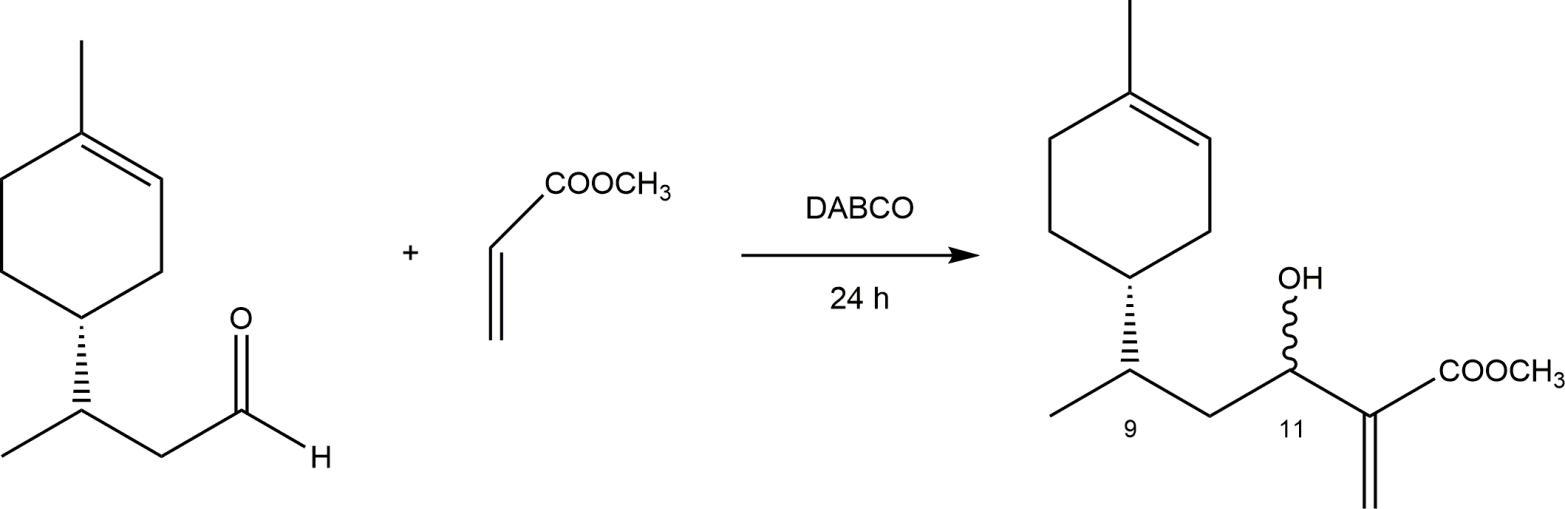
MBH reaction between hydroformylated (*R*)-limonene and methyl acrylate.

Several attempts were made to obtain adduct 1, derived from methyl acrylate, yielding an average of 32% (Scheme 4). The optimized conditions involved a reaction temperature of 40°C for 24 h, with no significant improvement observed upon extending the reaction time.

It was also found that a molar ratio of 3.5 : 1 (methyl acrylate to (*R*)-limonene) is optimal, as using a higher excess of methyl acrylate does not improve the reaction yield, while lower amounts significantly slow down the reaction rate.

The product structure was confirmed using several techniques, including elemental analysis, IR spectroscopy and ¹H NMR and ¹³C NMR spectroscopy, as detailed in §2 and the electronic supplementary material.

In the IR spectrum of the adduct, characteristic bands support the proposed structure. A broad and intense O–H stretching band appears at approximately 3500 cm⁻¹, consistent with the presence of an alcohol group. Additional bands include those near 3000 cm⁻¹ (C–H stretching of alkenes), 1720 cm⁻¹ (C=O stretching of esters), 1670 cm⁻¹ (C=C stretching of tri-substituted alkenes) and 1650 cm⁻¹ (C=C stretching of a vinylidene group), all of which support the successful formation of the desired compound.

The ¹H NMR and ¹³C NMR spectra further corroborate the proposed structure. Notably, the MBH reaction introduces a new stereogenic centre at carbon 11, resulting in the formation of four diastereomers. This is confirmed by the ¹³C NMR spectrum, in which several signals appear quadruplicated.

**Scheme 5.**
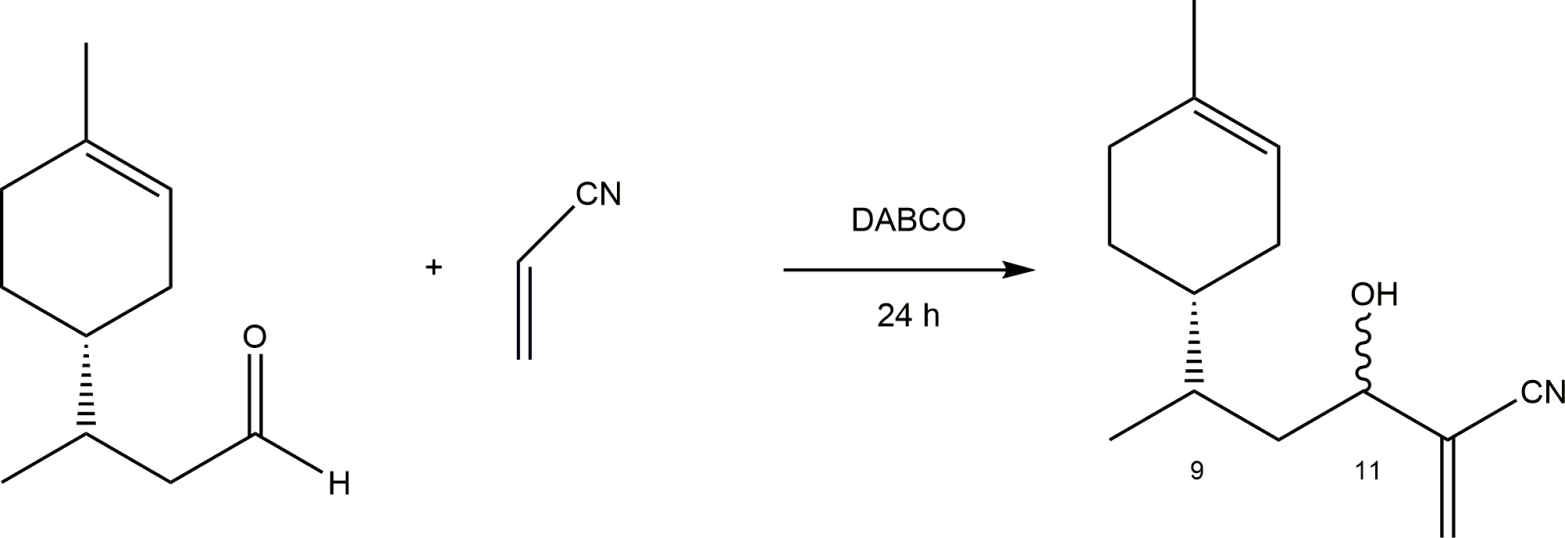
MBH reaction between hydroformylated (*R*)-limonene and acrylonitrile.

For the synthesis of adduct 2 (with acrylonitrile, Scheme 5), the initial reaction conditions—reaction time, temperature and molar ratio—were maintained. However, the outcome was significantly improved, with an average yield of 95%. The reaction tolerates a wide range of starting material quantities, from at least 0.300 g to 2.50 g (1.81 × 10⁻³ to 1.51 × 10⁻² mol). Increasing the amount of acrylonitrile does not substantially affect the reaction time.

As with adduct 1, adduct 2 was characterized using the same set of techniques.

In the infrared spectrum, in addition to the bands previously discussed, the main distinguishing feature is a weak and sharp absorption band at 2920 cm⁻¹, characteristic of C≡N stretching. This confirms the presence of a nitrile group in the product and is consistent with the proposed structure.

The ¹H NMR and ¹³C NMR spectra showed results similar to those of adduct 1, indicating the formation of four diastereomers without stereospecificity.

### 3.3. Comparison with related Morita–Baylis–Hillman adducts and discussion of characterization

The markedly different isolated yields observed for the two MBH products (adduct 1, 32%; adduct 2, 95%) can be rationalized by simple reactivity and steric considerations and are consistent with previous reports on MBH reactions of sterically demanding substrates. In our sequence, the hydroformylated (*R*)-limonene bears a congested allylic framework; coupling with an activated alkene therefore depends strongly on the electronic nature and steric profile of the Michael acceptor. Acrylonitrile, being a strongly electron-withdrawing and relatively small acceptor, provides faster conversion under our conditions and results in a high isolated yield for adduct 2. By contrast, methyl acrylate—although also an activated alkene—gave lower isolated yield (adduct 1), which may be attributed to slower C–C bond formation and to competing side processes (e.g. oligomerization of the Michael acceptor or partial decomposition of the aldehyde in traces of oxygen), phenomena previously noted for hindered terpene substrates in MBH chemistry. These observations qualitatively agree with recent methodological surveys and examples of MBH reactions that emphasize the strong influence of the acceptor and substrate sterics on yield [16,17,21].

Concerning characterization, the structural assignments for both adducts are supported by the collected spectroscopic data: IR bands characteristic of O–H and C=O (for the ester adduct) or C≡N (for the nitrile adduct), and the full set of ^1^H- and ^13^C-NMR resonances with integrals and multiplicities consistent with the proposed formulas (see §2 and the electronic supplementary material). The appearance of duplicated or quadruplicated ¹³C signals is interpreted as arising from the diastereomeric mixtures expected after formation of new stereogenic centres in the MBH step.

### 3.4. Biological tests

As described in §2, the evaluation of the biological activity of adducts formed from (*R*)-limonene derivatives was divided into three parts, each corresponding to the available methods for each class of microorganisms. Regarding the evaluation of antiprotozoal activity, the results obtained for adducts 1 (ester) and 2 (nitrile) are shown in table 1, where the IC_50_ values are presented in μg ml^−1^. The measurements were made in triplicate.

**Table 1.** Results of biological tests against protozoan species (IC_50_^a^ (μg ml^−1^)).

species	promastigotes, *Leishmania amazonensis*	epimastigotes, *Trypanosoma cruzi*
substances		
adduct 1	>500	475.0 ± 18.0
adduct 2	>500	>500

^a^ IC_50_ value represents the minimal concentration of a drug that is required for 50% inhibition *in vitro*.

The synthesized adducts are inactive against the tested protozoan species, especially in *Leishmania amazonensis*. Determining the IC_50_, which is higher than the highest concentration tested, was impossible. For tests performed with samples containing *Trypanosoma cruzi*, it was possible to decide on the IC_50_ for adduct 1 (475.0 μg ml^−1^). However, its value is still considered too high compared with the other classes of microorganisms evaluated in this work.

For the second class of tested microorganisms (some species of bacteria and fungi), the situation is similar: adducts 1 and 2 are also not active against such species even though it was possible to determine the range in which the BMC of adduct 1 (ester) is located for *Staphylococcus aureus*. The results obtained are shown in table 2.

**Table 2.** Results of biological tests against fungal and bacterial species (μg ml^−1^).

microorganisms	*S. aureus* BMC^a^	*E. coli* BMC^a^	*C. albicans* FMC^a^	*C. tropicalis* FMC^a^
substances				
adduct 1	250–1000	>1000	>1000	>1000
adduct 2	>1000	>1000	>1000	>1000

^a^ BMC (bactericidal minimal concentration) and FMC (fungicidal minimal concentration) are the minimum concentration of an antibacterial or antifungal agent required to kill a bacterium or fungus, respectively.

Finally, the possible antitumour activity of such adducts was also evaluated, and here, undoubtedly, are the most promising results of this work. Table 3 lists the carcinoma species tested, along with the respective CC_50_ values for each compound.

**Table 3.** Results of the evaluation of antitumour activity (CC_50_^a^ (μg ml^−1^) ± s.d.).

cell lines	HT-29^b^	PC-3^c^	HeLa^d^	HACAT^e^
substances				
adduct 1	211.4 ± 49.1	48.0 ± 2.6	37.9 ± 6.7	53.3 ± 3.1
adduct 2	353.0 ± 10.4	423.3 ± 20.8	351.7 ± 12.6	327.5 ± 53.0

^a^ CC_50_ is the concentration of test compounds required to reduce cell viability by 50%.

^b^ HT-29 (colon/intestinal carcinoma).

^c^ PC-3 (prostate carcinoma).

^d^ HeLa (cervical carcinoma).

^e^ HACAT (normal cell, human keratinocytes).

The first observation from the results above is that, regardless of the cell line analysed, adduct 1 (ester) is always more active than adduct 2 (nitrile), sometimes by a factor of 9.

The activity of adduct 1 for cervix carcinoma (HeLa) is the most promising result of the tests performed here. This value enables future studies on this adduct, including its potential adverse effects on the organism and its *in vivo* application. In this view, some considerations must be made. First, by evaluating CC_50_ values for normal human keratinocytes (HACAT) relative to the tested carcinomas, it is possible to observe some cytotoxicity of the synthesized adducts, that is, the safety margin of the growing inhibition of the carcinomas. The relationship to healthy cells is negligible, as the CC_50_ values are of the same order of magnitude.

Finally, comparing the values obtained in this work with those in the literature, there is considerable potential for the synthesized adducts. For adduct 1, the most promising in this work, although not as active against HT-29 cell lines as the compounds studied by Martinez [33] and Riva *et al*. [34], it shows similar activity to PC-351 strains, indicating a possible line of future research. Besides, although adduct 2 was not active against cancer cells but was produced in high yield, it can be easily transformed into the corresponding ester by applying the Pinner reaction [35] which opens new paths for this research.

## 4. Conclusion

The main objective of this work, to verify the viability of the hydroformylation reaction followed by the MBH reaction and to assess the bioactivity of such products, was successfully achieved. The results show the potential of this new series of derivatives from this source, which is so common in our country. After this work, a new class of products derived from a natural, abundant and cheap source is now open. In addition, the potential of such derivatives as possible alternatives for fighting tumour cells is encouraging and motivating for future work on the sequence of hydroformylation followed by MBH reaction, starting from molecules found in nature. Adduct 1 showed better antitumour activity, but its synthesis exhibited low conversion. Otherwise, adduct 2 can be produced in high yield, allowing the ester to be easily prepared from the nitrile via the Pinner reaction. Besides this derivatization, the following steps of this work will be the performing of the same sequence of reactions for other terpenes and other coupling agents in order to produce new compounds in which bioactivity is probable. Another issue is the enantioselective synthesis of such adducts, something of great importance and interest for the pharmaceutical industry, and it should be verified if any diasteromer is more effective against a specific target.

In summary, the sequential hydroformylation of (*R*)-limonene followed by MBH coupling provided two new MBH adducts obtained from methyl acrylate (adduct 1) and acrylonitrile (adduct 2). Both compounds were characterized by ¹H- and ¹³C-NMR and IR spectroscopy, confirming the expected structures. The synthetic sequence demonstrates the feasibility of converting a renewable terpene into multifunctional products through atom-economic reactions under mild conditions.

Among the two adducts, the methyl acrylate derivative (adduct 1) exhibited the most promising antiproliferative activity *in vitro*, with lower CC₅₀ values against HeLa and PC-3 cell lines. By contrast, the acrylonitrile derivative (adduct 2) stood out for its high isolated yield (95%) and synthetic versatility, serving as a convenient intermediate for further functionalization, such as ester or amide formation. These complementary results highlight how the electronic and steric nature of the Michael acceptor influences both yield and biological profile.

Overall, this study establishes a sustainable, straightforward route to bioactive MBH adducts from terpene feedstocks. Future work will focus on expanding the substrate scope to other terpenes and acceptors, and optimizing reaction conditions to improve both yield and stereochemical control.

## Data Availability

Data are supplied as electronic supplementary material [36].
